# P02.45. Description of naturopathic practice from clinical administrative data on 400,000 visits in academic clinics

**DOI:** 10.1186/1472-6882-12-S1-P101

**Published:** 2012-06-12

**Authors:** C Calabrese, E Oberg, S Chamberlin

**Affiliations:** 1 Naturopathic Physicians Research Institute, Portland, USA; 2 Bastyr University, Kenmore, USA; 3 National College of Natural Medicine, Portland, USA

## Purpose

Because naturopathic physicians may represent an underutilized public health resource, there is need to comprehensively characterize the care they provide to understand NDs’ role in healthcare systems. Characterization also provides preliminary data for clinical research and quality improvement initiatives. Descriptive data are efficiently sourced from clinical administrative records in computerized practice management systems used in most naturopathic clinics.

## Methods

Data collection was from the 7 accredited academic naturopathic clinics in North America, which are some of the largest and which operate diversely in different jurisdictions. Four institutions provided patient-level data and three provided summary data. We obtained records or reports for 2006-10 on the numbers and demographics of new/returning patients, visits/dates, diagnostic/procedure codes, direct costs billed and payment sources. After multi-institutional IRB approval, patient-level data was de-identified on site by the study team per HIPAA guidelines, reviewed and vetted for accuracy with clinic staff, then aggregated and harmonized to create a closed analyzable dataset. Parallel summary data from the other 3 institutions for comparisons provide an overall view of care provided. Analysis consisted of descriptive statistics and comparison of variables of interest across clinics by appropriate methods.

## Results

Preliminary analysis reveals broad ranges of gender, age, diagnoses and procedures with US clinics reliably reporting diagnostic and procedure codes. Fatigue, pain syndromes, infections and gastrointestinal complaints are the most frequently seen conditions. Visit patterns show a degree of continuity. Financial data are interpretable in some clinics with insurance claims filed as the most reliable component. Tables of results will be presented with some comparisons to other provider types. Analyses are not yet complete.

## Conclusion

Clinical administrative data is a rich source of health services information useful for little studied fields of healthcare. Characteristics of naturopathic services at academic clinics suggest a pattern of patients and diagnoses consistent with those of conventional primary care.

